# Environmental and pathological factors affecting the hatching success of the two northernmost loggerhead sea turtle (*Caretta caretta*) nests

**DOI:** 10.1038/s41598-023-30211-z

**Published:** 2023-02-20

**Authors:** Giudo Pietroluongo, Cinzia Centelleghe, Giuseppe Sciancalepore, Luca Ceolotto, Patrizia Danesi, Davide Pedrotti, Sandro Mazzariol

**Affiliations:** 1 Department of Comparative Biomedicine and Food Science, University of Padua, 35020 Legnaro, Italy; 2 Istituto Zooprofilattico Sperimentale delle Venezie (IZSVe), 35020 Legnaro, Italy

**Keywords:** Environmental impact, Diseases

## Abstract

In recent years, the report of loggerhead sea turtle (*Caretta caretta*) Mediterranean nesting range has expanded together with new records of nests becoming northward on the Italian coastline of the Tyrrhenian and Adriatic seas. These areas are characterized by intensive human activities, such as tourism, fishery, and marine traffic, all possibly involved in the influence of the use of coastal habitat by marine species. These anthropic threats, in addition to the natural ones and the changing environmental characteristics of the beach, may influence the growth of microorganisms causing hatching failures. Among microorganisms, fungal infection by the genus *Fusarium* (Link, 1809) is considered one of the main causes of globally declining sea turtle populations. In summer 2021, the two northernmost worldwide loggerhead sea turtle nests were monitored along the Northern Adriatic coastline (Veneto, Italy). These first records may potentially candidate this area as suitable for a large part of the loggerhead turtle’s life cycle and it could represent a minor sea turtle nesting area that, according to Prato and colleagues, remained unnoticed due to the lack of specific monitoring. Sea Turtle Egg Fusariosis (STEF) was deemed to have deeply compromised the hatching success of the northmost one. Climate change and anthropogenic impacts have been scored as one of the highest hazards to sea turtle health and could have played a role in the STEF development. Environmental changes, human activities, and emerging pathogens deserve the highest attention in terms of health research, and conservation management.

## Introduction

The loggerhead turtle, *Caretta caretta* (Linnaeus, 1758), is the most abundant sea turtle species in the Mediterranean Sea, representing a distinct population with specific demographic and genetic features^1,2^. Due to its conservation interest, it is listed in different international conventions for its protection (Habitat Directive 92/43/CEE; Convention on International Trade of Endangered Species; Bonn Convention; Bern Convention), and it has been deemed as “Vulnerable” in the International Union for Conservation of Nature (IUCN) Red List assessment.

In the Mediterranean Sea, every year loggerhead turtle nesting activities are reported with an estimation of more than 8000 eggs clutches annually laid^3^, mainly located in the easternmost region (i.e. Greece, Turkey, Cyprus, and Libya). The presence of nesting activities is also reported along the Italian coastlines^4^, with more frequent reports along the southern coastlines (i.e. Calabria, Sicily, Campania). In recent years, the report of loggerhead turtle nesting activity has increased with a total of 244 nests in 2021 in Italy (tartapedia.it), together with an expansion of the nesting range in the Mediterranean Sea^2^, and with new records of nests northward the Tyrrhenian and Adriatic coastlines^5,6^. These areas are characterized by more intensive human activities, such as tourism, fishery, and marine traffic, all possibly involved in the influence of the use of coastal habitat by marine species^7–12^. These anthropic activities, in addition to the natural threats, could impair egg-laying, embryotic development, carapacial abnormalities, and hatchlings survival on the beach^13^. For instance, during the embryotic stages, the hatchling success could be affected by a combination of factors such as human disturbance (i.e. beach cleaning, tourist activities), pollution, depredation, beach erosion, sediment granulometry, plant root invasion, excessive rainfall, tidal inundation, gas flow, salinity, humidity, and pathogenic infections^14–17^. All these factors may influence the physical parameters crucial for healthy embryonic development^18^ and the growth of microorganisms^15^, such as fungi and bacteria, reducing the hatchling success and causing embryonal death. Among microorganisms, fungal infections by the genus *Fusarium* (Link 1809) is considered one of the main causes of globally declining turtle populations^15,19,20^ also known as Sea Turtle Egg Fusariosis (STEF). *Fusarium spp.* members, including those belonging to the *F. solani* species complex (FSSC) and *F. oxysporum* species complex, have been isolated from the eggshells and embryonic tissue of failed sea turtle eggs for decades, with several hypotheses concerning their ecological role ranging from decomposition to pathogenicity^21^. Approximately 75% of fusariosis is caused by members of the FSSC, two closely related fungal species, *F. keratoplasticum* and *F. falciforme*^22^. They have been isolated from undeveloped eggs and embryos and deemed to be the causative agents of STEF^15,23,20^ causing mass mortalities in natural and relocated nests of the loggerhead turtles worldwide^15^.

The present study reports the case of the two northmost loggerhead turtles nesting sites reported to the best of our knowledge, which occurred along the northern Adriatic coastline (Veneto, Italy) in the summer of 2021. Furthermore, it describes how STEF has deeply compromised the hatching success of the northernmost one.

## Results

Nests’ characteristics and reproductive output are described in Table 1. In Nest 1, rainfalls and tides were constantly monitored to promptly face flooding of the area by permanent barricades and temporary sheltering. Collected data compared to the 2021 pluviometric data from the Regional Environmental Protection Agency confirmed an increase in rainfalls (+ 32%) in Jesolo compared to the period 1993–2020.

**Table 1:** Nest characteristics and reproductive output of the two nests.

	Nest 1	Nest 2
Nest characteristics		
Coordinates	45.498279 N, 12.629747 E	44.953619 N, 12.536725 E
Incubation period	68	ND
Distance from the shore (m)	2–50	20.25
Beach slope	0.9%	1.4%
Nest chamber depth (cm)	39	56
Depth of the first egg (cm)	23	39
Nest chamber width (cm)	24.5	36
Mean temperature	25° C	ND
Mean humidity	72.6%	ND
Granulometry mesh 0.250 mm	89.7%	90.2%
Nesting substrate	Sand	Sand
Reproductive outputs		
Total number of eggs	80	90
Hatched eggs	9	82
Eggs with yolk	25	7
Unhatched eggs	43	1
Eggs with embryos	1	1
Pipped eggs	2	0
Dead hatchlings	0	1
Hatching success rate	11.25%	91.1%
Hatchling emergence success rate	11.25%	ND
Mean hatclings CCL (cm)	4.4	4.6*

Nest 1 – Jesolo Lido, Nest 2 – Scano Boa.

*Calculated only in 2 hatchlings found.

**Figure 1:**
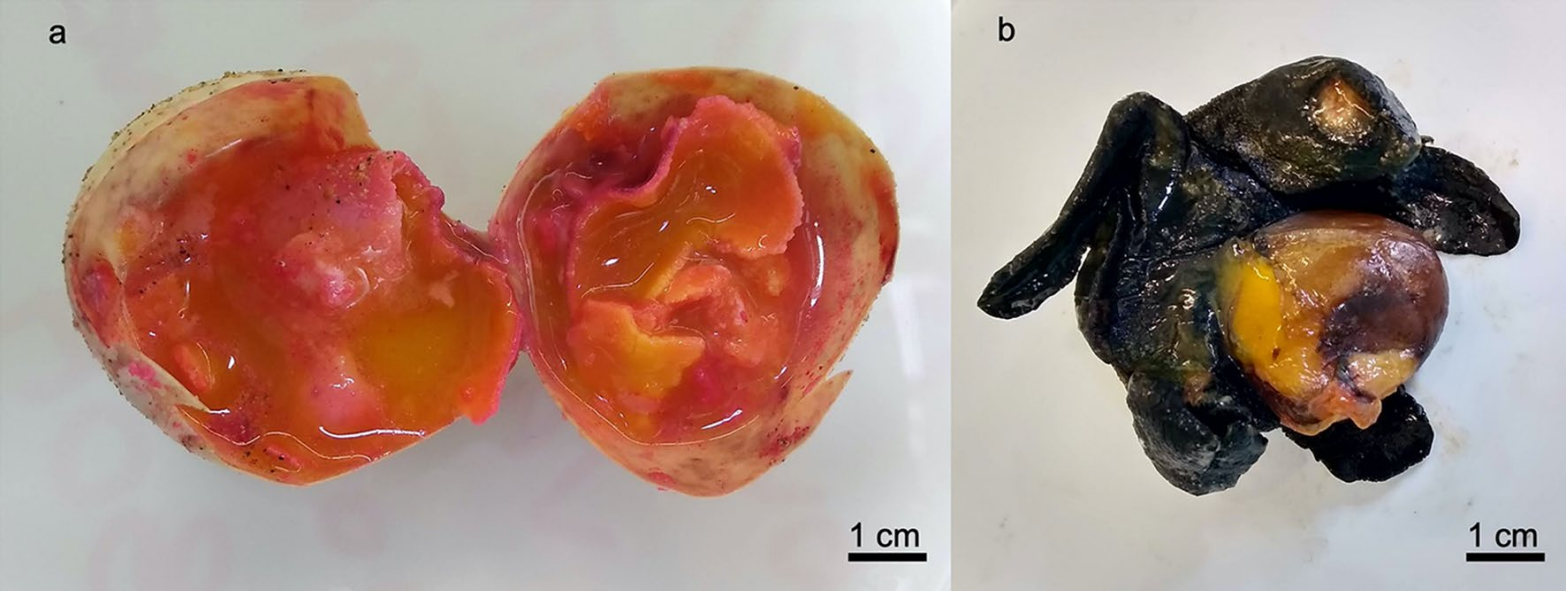
Nest 1 gross appearance. Macroscopical aspect of one unhatched egg (**a**) and one embryo (**b**) with evident signs of *F. oxysporum* infection. Scale bar = 1 cm.

During the inspection of Nest 1, 43 unhatched eggs, 1 embryo and 2 pipped eggs showed gross evidence consistent with fusariosis, including abnormal pink discoloration and incomplete development (later stage 23–31) (Fig. 1). No carapacial abnormalities were recorded on the hatchlings nor on the unhatched turtles. No additional gross findings were reported during the post-mortem examination of collected embryos.

The affected eggs were located at the periphery of the clutch, while the hatched eggs occupied the core. Eggs with yolk without any and the hatched eggs (25) occupied the deeper layer and the core of the incubation chamber respectively.

**Figure 2:**
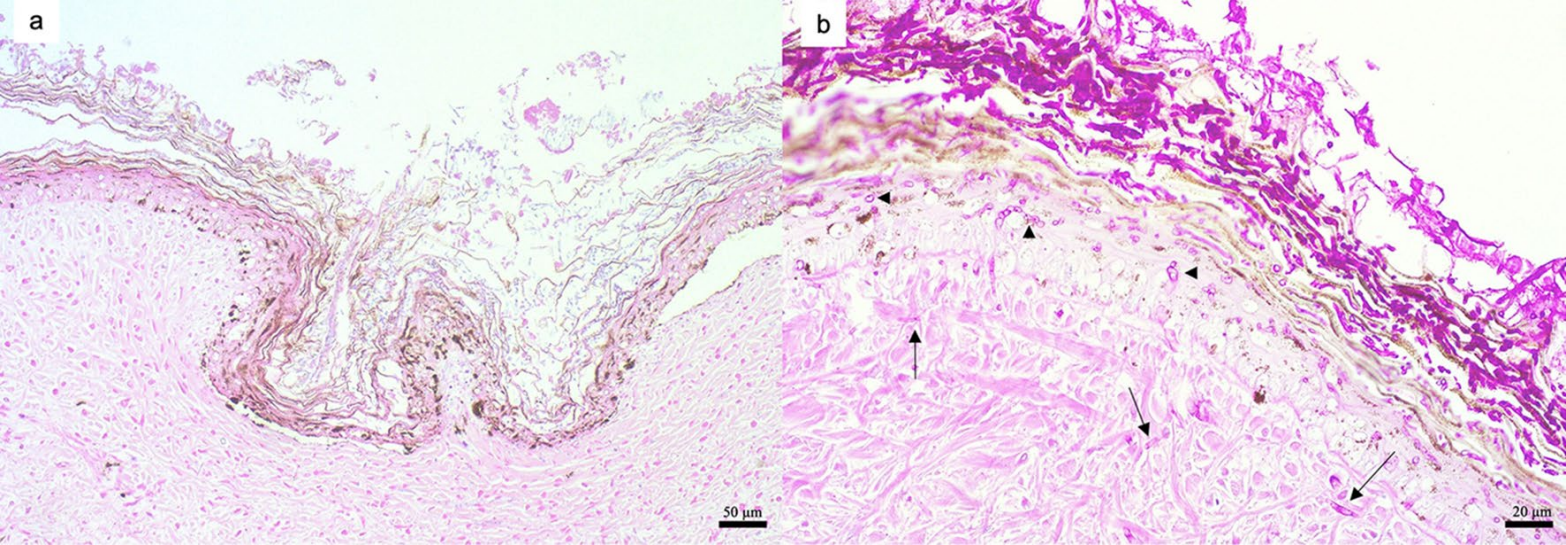
Nest 1 microscopical findings. Histological appearance of a section of an embryo’s skin (**a**) affected by multifocal degeneration of epidermal cells and orthokeratotic hyperkeratosis. (**b**) PAS stain underlined the presence of myriads of hyaline branched septate hyphae, 2 to 7 μm in diameter with thin, predominantly parallel wall attached to the skin, embedded within the epidermal layers (arrowhead) and in the superficial dermis (arrow). (**a**): hematoxylin and eosin staining, scale bar = 50 µm; (**b**): PAS staining, scale bar = 20 µm.

The microscopic examination of embryos revealed mild to moderate cellular degeneration in all the layers of the epidermis, mildly affecting also the level of the basement membrane and the superficial dermis, which appeared edematous and with loosely arranged collagen fibers. A severe diffuse thickening of the keratin layer with preserved keratinocyte maturation (orthokeratotic hyperkeratosis) was also evident (Fig. 2a). No other histological and microbiological findings consistent with any viral and/or bacterial infection were noted during the examination of the embryos. PAS and Grocott’s methenamine silver stains revealed an extensive fungal growth with myriads of yeasts and hyphae attached to the eggshell and the skin of the developing carapace, intermingled within the epidermal layers and, in less number, also in the dermis (Fig. 2b). The numerous hyaline hyphal elements were characterized by septation, acute-angle dichotomous or random branching, constrictions at branch points, and parallel thin walls, consistent with *Fusarium spp*. No evident inflammatory infiltrate was associated to these findings.

Fungal culture tested positive for filamentous fungi, growing from both eggshells, embryo, and yolk showing an intensive growth of *Fusarium* organisms. *F. oxysporum* was identified with similarity of 100% by blasting 28S rRNA sequencing in Genbank database. The mortality rate associated with *Fusarium spp*. was deemed to be 56.25%.

## Discussion

Marine biodiversity is affected by the changes related to global warming in different ways. Among all the species, sea turtles at any life stage are particularly susceptible to environmental changes and human activities^24^.

In the context of climate change, migration patterns, habitat use, sex ratio and embryonic development could be influenced by the effects of increased temperature^25^. Furthermore, these two nesting episodes added some concerns regarding the challenges in the management and monitoring of sea turtle nests in terms of interaction with human activities and health problems.

The two turtle nests hereby described, the episodes reported in Marche Region (Pesaro) in 2019, and the two in Liguria (Finale Ligure in 2021 and Levanto in 2022), as well as the confirmed increase in sea temperature in the Adriatic basin^26,27^ contribute to strengthen the hypothesis of the nesting activity expansion toward the northern coastline of the Western Mediterranean in the period 2010 – 2020^2,24^. In this context, Jesolo Lido and Scano Boa can be considered the northernmost nesting sites in the Mediterranean Sea ever monitored and, most likely, worldwide^28^. As shown by the data reported in Table 1, the two nests showed different hatching success with the lower results (11%) in the most urbanized location (Jesolo Lido) compared to the average (66%) reported in the Western Mediterranean^2^. Human presence and activities, such as the urbanization, the touristic preparation of the beach, and pollution, could have also compromised embryonal survival^24,29^.

The histopathological investigations reveal the presence of a *F. oxysporum* infection in Nest 1 which has been considered the cause of the lower hatchling success in this area. The infection was detected in the shallowest layer of the incubation chamber, while the hatched eggs occupied the deepest part of the core suggesting that they might have been protected by those more superficial. Moreover, Nest 1 compared to Nest 2 was shallower, with a different substrate composition, a lower slope, and a more intense impact of the tide, hence, these factors may have also influenced the hatchling success. No particular differences were reported on the hatchlings size also due to the few numbers of hatchlings monitored in Nest 2.

In marine ecosystems, the prevalence of infectious diseases caused by fungi has dramatically increased during the past two decades, likely due to the transmission of emerging pathogens into new environments and the rapid rate of global climate change^30,31^. Among these, *F. solani* has been recognized as the most frequent fungus in sea turtle mycotic diseases, and it is normally isolated and referred to as a “species complex” including more than 60 phylogenetic species^15^. These fungi can be found in the turtles’ digestive system and they could colonize eggs during their deposition, but they have also been reported on floating particles of plant tissues, silt, and plastics in the ocean which are carried by wind and currents to the beaches where the turtles lay their eggs^32^. Environmental factors can help the mycotic dissemination on failed eggs: the disease incidence and mortality are strongly affected by tidal inundation and sand with a high percentage of silt and clay^23^. Nest 1 area is a heavily urbanized beach with a large tidal range as shown by the distance of the nesting area from the shore (from 2 to 50 m depending on the tide) and a gentle slope artificially created by beach nourishment using sand from inland areas of riverine origin, including silt, clay and organic material to cope with the coastal erosion. Beach nourishment activities, which are constantly performed from early spring in the area of Nest 1, may also have influenced on hatchling success^33–29^. Also, the cleaning constantly performed using mechanical systems could have influenced the hatching success by inducing a shallower deposition and reducing the nest dimensions due to soil compaction, as evident by comparing the two different beaches in Table 1^35^. The aforementioned environmental factors (i.e. sand composition with organic matter, small and superficial nest chamber, tidal occurrence), besides being associated to a lower hatchling success, could have also influenced the development and diffusion of FSSC^36^ which has been globally recognized as a potential global threat to sea turtle eggs^37^. On the opposite side, Nest 2 occurred in a wilder area without human activities ongoing and showed a clearly higher hatching success rate (89%).

In conclusion, climate change and anthropogenic impacts have been scored as one of the highest hazards to sea turtle health and need the highest attention in terms of research and conservation management^38^. Additionally, identifying potential pathogens threatening endangered sea turtle species, also influenced by global warming and human activities, is crucial for developing conservation plans. The results of the present study contribute to the recent hypothesis of an expansion of the loggerhead sea turtle nesting area occurring in the Mediterranean basin, likely due to global warming^2,24^.

These first recordings of nesting activity and the historical data on loggerhead turtles^39^ may potentially candidate the Northern Adriatic Sea as a suitable area for large part of the loggerhead turtle’s life cycle, and it could represent a minor sea turtle nesting area as defined by Prato and colleagues (2022), which could have remained unnoticed due to the lack of specific monitoring^4,40^. The expansion in highly urbanized areas, with several anthropic activities having a negative impact on hatchling success, requires wider monitoring coverage as a priority action for sea turtle conservation. To cope with this possible menace, a dialogue between economical and conservation stakeholders should be focused on a management plan ensuring the coexistence of sustainable economic activities and the conservation of endangered species. These plans should include: (1) development of nest suitability models, real-time monitoring, nest protection, and inspection to cope with the negative effects of anthropic activities; (2) effective management strategies for the control of emerging diseases including their epidemiology^23^; and (3) the possible application of relocation practice, even if it remains unclear whether such an approach increases pathogens contamination or carry, hence the risk of FSSC infection and mortality in sea turtle eggs^41^.

## Materials and methods

### Nesting sites and monitoring

The present study was performed along the Veneto coastline, in the northwestern Adriatic Sea, Italy. The Northern Adriatic Sea is considered an important foraging and overwintering habitat, especially for subadult and adult female loggerhead turtles^39^. Sandy beaches and shallow waters characterized the coastal area, where no sea turtle nesting activity has ever been recorded. In July 2021, sea turtle nesting activity was reported by locals in Jesolo Lido (Venezia), one of the major touristic beaches in Italy with 1.491.296 and 1.738.396 tourists respectively in July and August 2021 (Statistical Data of Tourism from Veneto Region). After examination and confirmation of the nest presence (Nest 1), the area was protected and systematically monitored. Environmental parameters (temperature, humidity, weather, and tidal trends) were continuously monitored to prevent or face any flooding and record possible environmental influences on the hatchling success. Weather conditions were compared with historical data from the Regional Environmental Protection Agency (ARPAV)^42^.In September 2021, the presence of hatchlings was reported also in Scano Boa (Rovigo), a wild beach included in the Parco Regionale Veneto del Delta del Po (WDPA 178,945; EUAP 1062). This nest (Nest 2) was examined and confirmed too. After 13 days of the conclusion of the hatching period, no hatching, and a total of 81 days from the nesting of Nest 1, both nests were inspected, according to the Italian ministerial guidelines^43^. The main characteristics and reproductive outputs were recorded.

### Nest sample collection and characteristics

During the inspection, samples of sand were collected for granulometry analysis and to assess further characteristics of both nests^44^. All the eggs of the nests were collected to assess any possible changes in their color as a possible indication of the presence of microorganisms^15^. Furthermore, the eggs were examined to determine the development stage and the external morphological characteristics; those still unhatched were finally opened to assess the presence of any dead embryo according to the description by Miller and colleagues^45^.

### Post-mortem investigations

Gross inspection has been performed on embryos in order to assess any pathological change according to standardized post-mortem procedures^46,47^.

Eggshell, embryo and yolk samples from all unhatched eggs both with macroscopic changes and from 3 specimens (1 embryo and 2 pipped eggs) without apparent macroscopic lesions were collected for additional analysis. The samples from eggshell, embryo and yolk for microscopic examination were fixed in 10% formalin, embedded in paraffin, sectioned at 5 µm and mounted onto TOMO Adhesion Microscope Slides (Matsunami Glass), stained with hematoxylin and eosin (HE) using a semi-automatic histo-stainer (Leica Autostainer XL, Leica Biosystems Nussloch GmbH). Additionally, periodic acid–Schiff (PAS) and Grocott’s methenamine silver stain were performed in case of suspected fungal infections. Additionally, standard bacteriological examinations were routinely performed on the above-mentioned fresh eggshell, embryo, and yolk samples.

### Fungal culture and *Fusarium* species identification

Since gross pathological findings of the Nest 1 were consistent with Fusariosis, samples from eggshell, embryo and yolk were cultured on Sabouraud dextrose agar and incubated at 25 °C for at least 10 days. All fungal colonies identified morphologically belonging to the *Fusarium* genus were further molecularly processed for identification at species level. DNA extracted from *Fusarium* colonies was amplified by using SYBR Green Real-Time PCR (rtPCR) with a set of primers targeting a portion of the D1-D2 domain of the 28S rRNA gene using primers NL1/NL4 as previously described^48^. All amplicons were sequenced for fungal identification by using Blast in the GenBank database.

### Animal ethics

All methods were carried out in accordance with relevant Italian ministerial guidelines and regulations (*Linee guida per il recupero, soccorso, affidamento e gestione delle tartarughe marine ai fini della riabilitazione e per la manipolazione a scopi scientifici. ISPRA-MATTM, Manuali e Linee Guida 89, 2013*).

## Data Availability

All data generated or analyzed during this study are included in this published article.
